# Multi-view graph-based interview representation to improve depression level estimation

**DOI:** 10.1186/s40708-024-00227-w

**Published:** 2024-06-04

**Authors:** Navneet Agarwal, Gaël Dias, Sonia Dollfus

**Affiliations:** 1 UNICAEN, ENSICAEN, CNRS, GREYC, Normandie Univ, 14000 Caen, France; 2 Service de Psychiatrie, CHU de Caen, 14000 Caen, France; 3 UNICAEN, ISTS, GIP Cyceron, Normandie Univ, 14000 Caen, France; 4 UNICAEN, UFR de Médecine, fédération hospitalo-universitaire-FHU A2M2P, Normandie Univ, 14000 Caen, France

**Keywords:** Graph-based interview transcript representation, Multi-view architectures, Similarity graphs, Keyword correlation graphs, Insight generation, Automatic depression level estimation

## Abstract

Depression is a serious mental illness that affects millions worldwide and consequently has attracted considerable research interest in recent years. Within the field of automated depression estimation, most researchers focus on neural network architectures while ignoring other research directions. Within this paper, we explore an alternate approach and study the impact of input representations on the learning ability of the models. In particular, we work with graph-based representations to highlight different aspects of input transcripts, both at the interview and corpus levels. We use sentence similarity graphs and keyword correlation graphs to exemplify the advantages of graphical representations over sequential models for binary classification problems within depression estimation. Additionally, we design multi-view architectures that split interview transcripts into question and answer views in order to take into account dialogue structure. Our experiments show the benefits of multi-view based graphical input encodings over sequential models and provide new state-of-the-art results for binary classification on the gold standard DAIC-WOZ dataset. Further analysis establishes our method as a means for generating meaningful insights and visual summaries of interview transcripts that can be used by medical professionals.

## Introduction

Depression is a serious mental disorder that affects millions of people worldwide. According to statistics from the World Health Organization (WHO),^1^ approximately 5% of adults worldwide experienced depression in 2019, amounting to an estimated 280 million people. We expect a curve of increasing estimates as a consequence of recent global events including the health crisis that caused widespread unemployment, stress and feeling of isolation [1]. This increased need for mental healthcare can lead to significant burden on the current system with the number of patients reaching beyond the capacity of available medical resources in some cases. In the Ain region of France for example, the supply of psychiatric care is half the national average with 9 psychiatrists for 100,000 inhabitants.^2^ Advancements in the field of machine learning have allowed researchers to develop automated tools that can help reduce stress on the healthcare system, with particular attention being paid to automated depression estimation.

Evaluation of depression is a challenging problem with patient-therapist interviews being the common practice within the medical community for analyzing a person’s mental health. Psychiatrists use these dialogues as a means to examine different aspects of a patient’s life (including work, living situations, family, relationships, etc.) in order to ascertain their mental state. Complementary to these interviews, different screening tools have also been defined that quantify a person’s psychological state. Among these tools, the Patient Health Questionnaire (PHQ-8) is considered a valid diagnosis and severity measure for depressive disorders [2] and represents self-assessment scores for eight indicative symptoms of depression: loss of interest, feeling of depression, sleeping disorders, feeling of tiredness, loss of appetite, feeling of failure, lack of concentration and lack of movement.

Throughout the literature, different strategies have been proposed for automated estimation of depression. These approaches aim to infer the screening tool score (PHQ-8 score) based on patient-therapist interviews conducted in a clinical setting. Multi-modal architectures combine inputs from different modalities [3, 4]. Multi-task architectures simultaneously learn related tasks [4, 5]. Gender-aware models explore the impact of gender on depression estimation [6, 7]. Hierarchical models process transcripts at different granularity levels [8, 9], Attention models integrate external knowledge from mental health lexicons [9], feature-based solutions compute multiple multi-modal characteristics [10]. Multi-view model treats the input transcript as combination of two views in order to incorporate the discourse structure [11]. Graph-based architectures process non-linear structures within the conversation using graph neural networks (GNN) [12–14]. Symptom-based models treat depression estimation as an extension of the symptom prediction problem [15]. Domain-specific language models have been built [16] and large language models are prefix-tuned to automate depression estimation [17].

Despite this extensive list of initiatives, input representation remains a relatively unexplored research direction. Most researchers utilize a sequential encoding of the interview transcripts without considering the inherent non-linear structure of conversations. Within this paper, we explore graph-based representations of patient-therapist interviews and study their impact on the depression estimation task. Our primary focus is directed towards sentence similarity graphs and keyword correlation graphs [18], which highlight distinctive features across varying levels of granularity within the data. These graph structures not only highlight complex interactions within the discourse but also provide a perspective that does not exist within sequential data models. Additionally, we expand upon the research conducted by Agarwal et al. [11], taking the concept of multi-view from neural network architectures and applying it to graph-based input representations. As such, we propose to take into account the inherent relation between questions and answers within interview transcripts. We show steady improvements in performance by combining the multi-view concept with input graphs, thus reinforcing the validity of the multi-view idea. Finally, we demonstrate that visual representation of graph structures can serve as a rapid visual synopsis of the conversation while providing valuable insights for healthcare experts. A wide range of experiments have been endeavoured over the gold standard Distress Analysis Interview Corpus-Wizard of Oz (DAIC-WOZ) dataset [19] for the binary classification problem, which shows advantages of using graph-based representations over sequential input and combining them with the multi-view approach. Our best model provides new state-of-the-art results over the DAIC-WOZ with a macro-F1 score of 81% for the binary classification task.

## Related work

Different architectures and strategies have been used throughout the literature to train automated models for depression estimation based on patient-therapist interviews. One promising research area is to leverage inputs from different modalities into one learning modal. Qureshi et al. [4] explore the possibility of combining audio, visual and textual input features into a single architecture using attention fusion networks. They further show that training the model for regression and classification simultaneously on the same dataset provides improvements in results. Ray et al. [3] present a similar framework that invokes attention mechanisms at several layers to identify and extract important features from different modalities. The network uses several low-level and mid-level features from audio, visual and textual modalities of the participants’ inputs. Another interesting approach aims at combining different tasks that share some common traits thus following the multi-task paradigm. Qureshi et al. [5] propose to simultaneously learn both depression level estimation and emotion recognition on the basis that depression is a disorder of impaired emotion regulation. They show that this combination provides improvements in performance for the multiclass problem as well as the regression of the PHQ-8 score. Building on the success of hierarchical models for document classification, different studies [8, 9] propose to encode patient-therapist interviews with hierarchical structures, showing boosts in performance. Xezonaki et al. [9] further extend their proposal and integrate affective information (emotion, sentiment, valence and psycho-linguistic annotations) from existing lexicons in the form of specific embeddings. Exploring a different research direction, Qureshi et al. [7] study the impact of gender on depression level estimation and build four different gender-aware models that show steady improvements over gender-agnostic models. In particular, an adversarial multi-task architecture provides best results overall. Along the same line, Bailey et al. [6] study gender bias from audio features as compared to [7], who target textual information. They find that deep learning models based on raw audio are more robust to gender bias than ones based on other common hand-crafted features, such as mel-spectrogram. Although most strategies rely on deep learning architectures, a different research direction is proposed by Dai et al. [10], who build a topic-wise feature vector based on a context-aware analysis over different modalities (audio, video, and text). Niu et al. [12] use graph structures within their architecture to grasp relational contextual information from audio and text modality. They propose a hierarchical context-aware model to capture and integrate contextual information among relational interview questions at word and question-answer pair levels. Along the same research direction, Hong et al. [13] use a graphical representation of the input that encodes word-level interactions within each transcript. They propose schema-based graph neural networks and use multiple passes of the message passing mechanism [20, 21] to update the schema at each node of the text graph. Burdisso et al. [14] define a more complex input graph structure that models the interactions between transcripts and a global word graph. They use an inductive version of graph convolutional networks (GCN) [22] and define *w*-GCN that mitigates the assumptions of locality and equal importance of self-loops within GCN. Milintsevich et al. [15] treat binary classification as a symptom profile prediction problem and train a multi-target hierarchical regression model to predict individual depression symptoms from patient-therapist interview transcripts. Agarwal et al. [11] highlight the importance of retaining discourse structure and define multi-view architectures that divide the input transcript into views based on sentence identities. The two views are processed both independently and co-dependently in order to account for intra-view and inter-view interactions. Building upon the success of language models in understanding textual data, Ji et al. [16] fine-tune different BERT-based models on mental health data and provide a pre-trained masked language model for generating domain-specific text representations. Lau et al. [17] further account for the lack of large-scale high-quality datasets in the mental health domain and propose the use of prefix-tuning as a parameter-efficient way of fine-tuning language models for mental health.

### Multi-view architectures

Within the context of depression estimation based on patient-therapist interviews, most research works disregard sentence types, patient or therapist input, and treat the input transcript as a sequence of unstructured sentences. Agarwal et al. [11] highlight the importance of retaining discourse structure and propose multi-view architectures that utilize sentence types as a means to incorporate the said structure into the learning process. Within multi-view architectures, interviews are considered as interactions between two perspectives, rather than being strictly dependent on sequential sentence structure. The input transcript is divided into two views based on sentence types, patient and therapist views, which are then treated both independently and co-dependently. Dedicated neural networks are used to learn transcript-level representations of individual views, with inter-view and intra-view interactions modeled using attention mechanisms. In particular, Agarwal et al. [11] propose a combination of self-attention and cross-attention mechanisms where different metrics are tested to reach high performance during cross-attention. More recently, the same authors propose in [23] a multi-head cross-attention mechanism that overperforms their original proposal. Overall results show the advantages of multi-view encoding over structure-agnostic sequential text representations.

### Keyword correlation graphs

Most representation learning methods model text as bag-of-words or as sequences of variable-length units, and are ineffective in capturing global features. *Keyword correlation graphs* (KCG) [18] represent documents as a weighted graph of topical keywords, and integrate global information into the input using learned topical knowledge. Within the KCG definition, topic modeling is used to learn a set of global topics that are used for extracting important keywords within each document. These topic models are trained on the entire training data, and hence encode corpus-level understanding of the text which is then incorporated within the input structure of individual transcripts. Each node within the graph represents a keyword, with sentences in the document assigned to the node they are most related to. The edges between the nodes indicate their correlation strength which is calculated based on pair-wise cosine similarity between corresponding sentence sets.

## Methodology

Graphs are discrete structures that provide a more intuitive way to not only capture the non-linear connections within conversations, but also define intricate input representations that do not exist in a linear setting. Based on the definitions of nodes and their interactions, different graph structures can highlight different aspects of the same input transcripts. In this paper, we experiment with *sentence similarity graphs*, focusing on local sentence level interactions, and *keyword correlation graphs*, highlighting global corpus level features within the input. We also extend the multi-view concept introduced by Agarwal et al. [11] to graph-based transcript representations. This extension aims to further highlight the different perspectives and their interactions within the input discourse. In particular, we not only use multi-view architectures within our network definition, but also apply it to the input representations and define graph structures depicting the two views. Based on the experiments of [11], the *MV-Inter-Att. (Mean)* configuration of multi-view architecture is used within our network definitions.

### Sentence similarity graphs

**Fig. 1:**
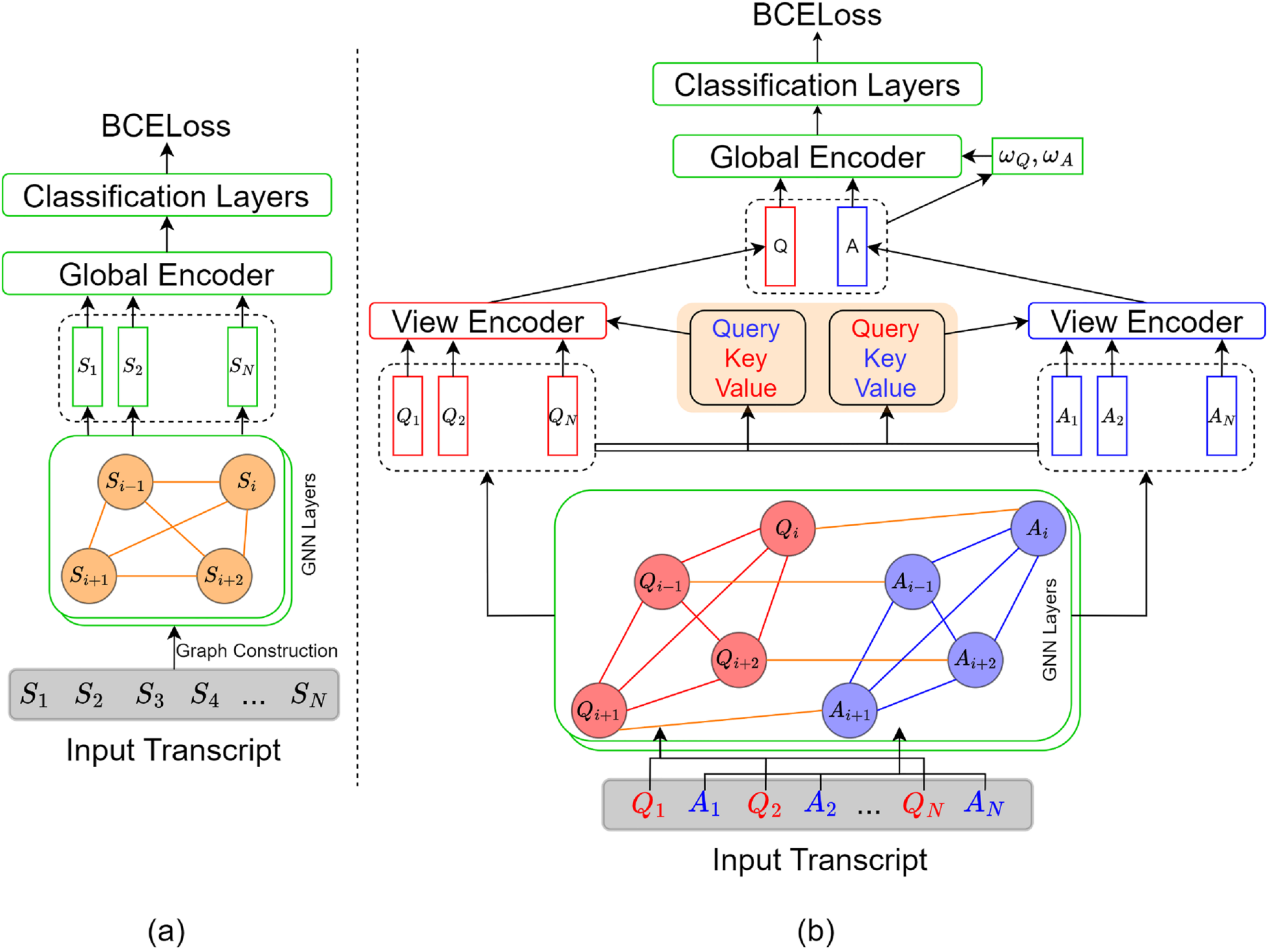
Configurations of sentence similarity graphs. Input color coding; red: therapist view, blue: patient view, orange: global nodes/cross connections, green: global network

Sentence similarity graphs are the most basic graphical representation of data that highlight sentence level interactions within text. Individual sentences form the nodes of the graph and edges are defined using cosine similarity between corresponding node embeddings. Within this context, we define two configurations that explore both generic definition of sentence similarity graphs and the multi-view infused interpretation. Figure 1 provides an overview of the graph structures and architectures used within this setting.

#### Similarity-baseline

We start with the generic definition of sentence similarity graphs where all sentences are treated equally irrespective of their identity (patient or therapist input). Edges are defined between all possible node pairs based on cosine similarity between corresponding node embeddings. Similarity threshold is applied to introduce sparsity into the graph structure with its value treated as an hyper-parameter during training. GNNs followed by multi-head attention and classification layers are used to process this graph structure as illustrated in Fig. 1a.

#### Similarity-MV

Keeping inline with the multi-view idea, we divide the transcript into patient and therapist inputs. Individual sentence similarity graphs are defined for the two views (highlighted with red and blue colors in Fig. 1b) encoding the intra-view interactions. Cross connections, represented by edges between the corresponding questions and answers (shown in orange) represent inter-view interactions within the input. GNNs are used to process the resulting graph structures and the learned sentence embeddings are used as sentence level encodings within the multi-view architecture. This configuration highlights the multiple perspectives and non-linear interactions within patient-therapist interviews.

### Keyword correlation graphs

**Fig. 2:**
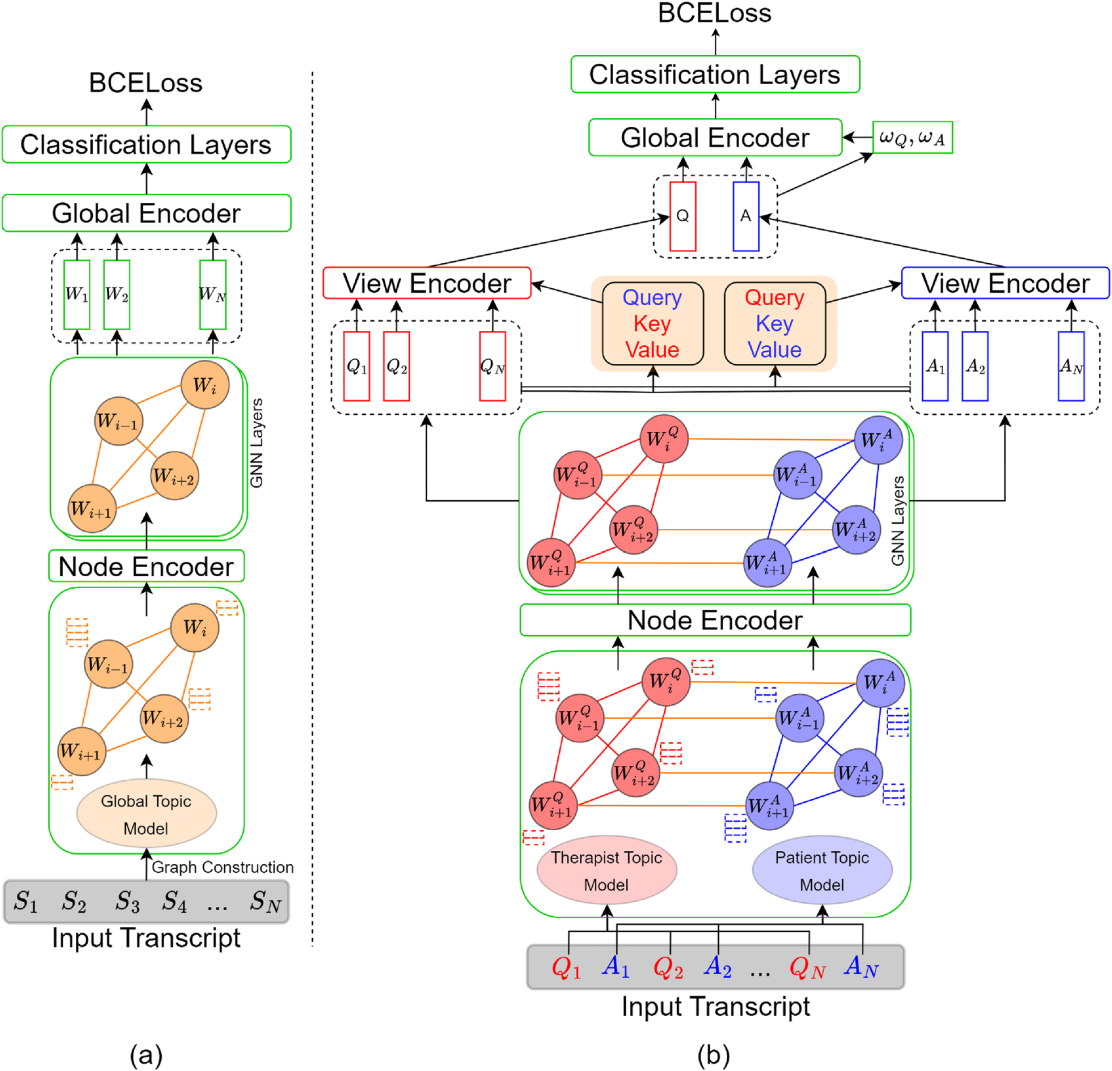
Different configurations of keyword correlation graphs. Input color coding; red: therapist view, blue: patient view, orange: global nodes/cross connections

Sentence similarity graphs, although good at representing local sentence level interactions within a transcript, are ineffective in capturing global features. Within the context of patient-therapist interviews, topics discussed within each transcript (like work, family, children, living situation, etc.) belong to a larger finite set of topics shared across all interviews. We propose to use keyword correlation graphs in order to combine transcript and corpus level knowledge and define each input as a graph of important topical keywords representing the transcript. Due to the semi-structured nature of interviews, psychiatrists typically discuss most of the relevant aspects of a person’s life within each interview. Consequently, to attain more distinct and differentiating topics, we employ sentence-level inputs to train our topic models instead of using transcript-level text. Non-negative Matrix Factorization (NMF) [24] is used as topic model and trained on a collection of all sentences within the training set with each sentence treated as an individual document. These models encode global corpus level topical knowledge and are used to infer word importance within each transcript, with 50 most important keywords used as nodes within the graph structure. *Node Encoder* layer, defined using multi-head attention architecture, is used to generate learned node embeddings by combining encodings of corresponding sentence sets. Keyword interactions are defined using average pairwise cosine similarity between their corresponding sentence embedding sets. As in the previous case, we also define multi-view inspired KCG architecture. Figure 2 shows an overview of the different configurations used within this context.

#### KCG-baseline

For the baseline configuration, we treat all sentences equally and train a single topic model on collection of all sentences within the training set. This global topic model represents combined topical knowledge from both therapist and patient inputs and is used to generate a single keyword correlation graph structure for each individual transcript. A neural network similar to *Similarity-Baseline* configuration is used to process the resulting graph. *Node Encoder* layer learns node embeddings by combining sentence encodings from the corresponding sentence sets.

#### KCG-MV

Although topics are shared across interviews, within this configuration, we explore the possibility that the two views of the data represent complementary topical information. Independent topic models learned from the two views can be used to highlight different aspects of the same interview. Topic model based on therapist’s questions represents the relevant aspects of patient’s life while patients’ topic model might be better suited for understanding their emotions and feelings. Within this configuration, transcripts are divided into patient and therapist inputs and individual NMF models are trained to learn topics within the two views. Dedicated keyword correlation graphs are generated using corresponding topic models to represent the two views. Cross connections within this configuration are used to study patients’ feelings with regards to different aspects of their life, and are defined based on presence of corresponding question and answer in the sentence sets of the nodes. A neural network architecture similar to *Similarity-MV* is used to process the resulting graph.

## Data

For our experiments, we use the Distress Analysis Interview Corpus-Wizard of Oz (DAIC-WOZ) dataset which is part of a larger corpus, the Distress Analysis Interview Corpus (DAIC) [25]. The dataset contains clinical interviews aimed towards psychological evaluation of participants for detecting conditions such as anxiety, depression and post-traumatic stress disorder. These interviews were collected with the goal of developing a computer agent that interviews participants to identify verbal and non-verbal signs of mental illness [26]. In particular, we use Wizard-of-Oz interviews from the dataset which were conducted by virtual agent Ellie, controlled by a human interviewer from another room. These interviews have been transcribed and annotated for a variety of verbal and non-verbal features. Along with the transcripts, the dataset also contains corresponding visual and audio features extracted from the interview recordings. Depression severity is assessed based on the PHQ-8 depression scale, and a score of 10 is used as threshold to differentiate between depressed and non-depressed participants. The dataset is divided into training, development and test sets containing 107, 35 and 47 interviews respectively. The dataset is biased towards lower PHQ-8 scores with almost 70% data points belonging to the negative class in case of binary classification (PHQ-8 score < 10) and only 4 instances with severe depression (PHQ-8 score > 20). Refer to Table 1 for more details.

**Table 1:** Number of interviews for each depressive class severity in the DAIC-WOZ dataset, distributed by train, validation and test sets

Depression severity	Data split		
	Train	Val.	Test
No symptoms [0..4]	47	17	22
Mild [5..9]	29	6	11
Non-depressed Total	76	23	33
Moderate [10..14]	20	5	5
Moderately severe [15..19]	7	6	7
Severe [20..24]	4	1	2
Depressed Total	31	12	14
Total	107	35	47

## Experimental setups

We use sentence-transformers [27], all-mpnet-base-v2 in particular, for generating sentence level text encodings. Adam optimizer with learning rate of $5\times 10^{-4}$ along with weighted binary cross entropy loss (BCELoss) have been used during training to account for class imbalance in data. Similarity threshold has been applied to introduce sparsity into graphs, and the value is treated as an hyper-parameter during training. Graph convolutional networks [28] are used to define GNN layers. All encoders, *global encoder*, *view encoder* and *node encoder*, are defined using transformer-based multi-head attention networks [29]. Cross-attention at *view encoder* level is also defined using transformer-based multi-head attention networks with inputs from the two views playing different roles among query, key and value as per requirement. Pytorch and Pytorch Geometric [30] frameworks are used for network definition and training.

## Results and analysis

Table 2 provides detailed results for all configurations. Best models are chosen based on macro-F1 scores on the development set and performance on both the development and test set are reported. Mean and standard deviation are calculated over 5 random initializations for all models. In order to establish a sequential baseline, we also include results by Agarwal et al. [23] who experiment with comparable neural network architectures applied to linear input configurations, i.e. without graph encodings. Figures prove that graph-based representation of transcripts provides better and more stable performance compared to sequential representation. In particular, *Similarity-MV* representation evidences best-performing results for 3 out of 4 evaluation metrics outperforming all other configurations considered in our research.

**Table 2:** Overall results over the DAIC-WOZ dataset

Configurations	Macro-F1		UAR		Accuracy		Precision (macro)	
	(Dev)	Test	(Dev)	Test	(Dev)	Test	(Dev)	Test
Linear-Baseline [23]	(0.79 ± 0.04)	0.75 ± 0.04	(0.78 ± 0.03)	0.75 ± 0.04	(0.82 ± 0.03)	0.80 ± 0.03	(0.82 ± 0.04)	0.77 ± 0.04
Linear-MV [23]	(0.77 ± 0.02)	0.80 ± 0.02	(0.76 ± 0.03)	**0.83 ± 0.02**	(0.80 ± 0.02)	0.82 ± 0.02	(0.79 ± 0.02)	0.79 ± 0.02
Similarity-Baseline	(0.71 ± 0.00)	0.77 ± 0.03	(0.70 ± 0.00)	0.77 ± 0.04	(0.76 ± 0.00)	0.81 ± 0.02	(0.75 ± 0.00)	0.78 ± 0.03
Similarity-MV	(0.76 ± 0.0)	**0.81 ± 0.01**	(0.74 ± 0.0)	0.82 ± 0.01	(0.79 ± 0.0)	**0.83 ± 0.01**	(0.78 ± 0.0)	**0.80 ± 0.01**
KCG-Baseline	(0.67 ± 0.03)	0.68 ± 0.01	(0.66 ± 0.03)	0.69 ± 0.01	(0.73 ± 0.02)	0.72 ± 0.01	(0.73 ± 0.04)	0.68 ± 0.01
KCG-MV	(0.66 ± 0.02)	0.76 ± 0.03	(0.65 ± 0.02)	0.74 ± 0.03	(0.72 ± 0.01)	0.81 ± 0.02	(0.72 ± 0.02)	**0.80 ± 0.02**

UAR stands for Unweighted Average Recall. We provide results for both development and test sets. The best model is chosen based on macro-F1 values over the development set and the best results over the test set are highlighted in bold. All models are run 5 times and average results and standard deviation are reported

**Fig. 3:**
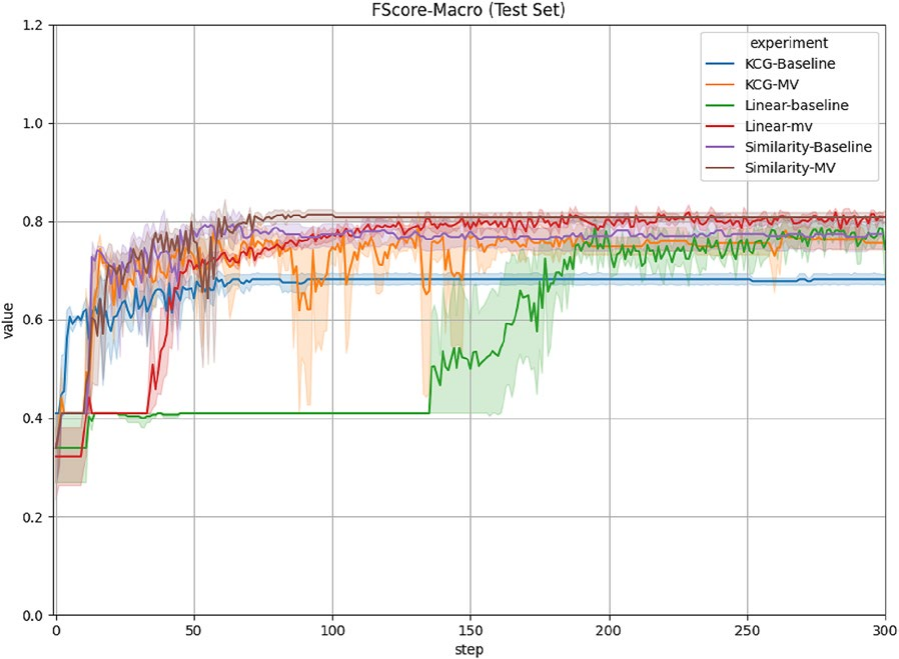
Plot of macro-F1 score distributed by epochs for different configurations on the test set

From Table 3, we further show that our best-performing model (*Similarity-MV*) provides new state-of-the-art results over the DAIC-WOZ dataset, outperforming recent initiatives including those relying on external knowledge (HAN+L [9]), different modalities (SVM:m-M &S [10]) or multi-target learning (Symptom Prediction [15]). Figure 3 also proves that our graph-based models not only provide state-of-the-art results but also have a stable learning curve, which is a desirable property for applications in the medical domain. Note that the reported results are taken directly from the original papers, and some related work surprisingly do not evidence results over the test split, such as HCAG and HCAG+T [12], although they highly perform on the development set. This might suggest a strong overfitting of the models.

**Table 3:** State-of-the-art results on DAIC-WOZ

Architectures	Modality	macro-F1		UAR	
		(Dev)	Test	(Dev)	Test
Raw Audio [6]	A	(0.66)	–	–	–
SVM:m-M &S [10]	T+V+A	(0.96)	0.67	–	–
HCAG [12]	T+A	(0.92)	–	(0.92)	–
HCAN [8]	T	(0.51)	0.63	(0.54)	0.66
HLGAN [8]	T	(0.60)	0.35	(0.60)	0.33
HAN [9]	T	(0.46)	0.62	(0.48)	0.63
HAN+L [9]	T	(0.62)	0.70	(0.63)	0.70
HCAG+T [12]	T	(0.77)	–	(0.82)	–
MV-IA-Mean [11]	T	(0.69)	0.73	(0.68)	0.72
Symptom Pred. [15]	T	(0.72)	0.74	–	–
**Similarity-MV**	T	(0.76)	**0.81**	(0.74)	**0.82**

T, V and A stand for Text, Visual and Audio modalities respectively

Best performance over the test set is highlighted in bold

### Sequential vs. graph-based input representation

Comparing linear and sentence similarity graph-based models, we see improvements with graphical representations for both *Baseline* and *MV* configurations. Specifically, *Similarity-Baseline* outperforms *Linear-Baseline* by 2.6% on macro-F1 score while *Similarity-MV* outperforms *Linear-MV* by 1.2% for the same metric. Overall, *Similarity-Baseline* outperforms *Linear-Baseline* for 4 out of 4 metrics while *Similarity-MV* evidences better results than *Linear-MV* for 3 out of 4 metrics. Furthermore, Fig. 3 plots macro F1 score against the step count for all architectures considered in this research. The plots show more stable convergence curves for graph-based models (*Similarity-MV* and *KCG-MV* in particular), which is a highly desirable property within healthcare applications. This is further supported by standard deviation values reported in Table 2. Our results highlight the benefits of using graph-based input representations, both in terms of predictive performance and stability of the proposed models.

### Baselines vs. multi-view architectures

Within our experiments, multi-view based representations steadily outperform the corresponding baseline configurations for both sentence similarity graphs and keyword correlation graphs. In particular, *Similarity-MV* outperforms *Similarity-Baseline* by 5% on macro-F1, 6% on UAR, 2% on Accuracy, and 2% on Precision (macro). Sentence similarity graphs focus on local sentence-level interactions where the context shared between the corresponding questions and answers plays an important role. This strong dependency between the two views restricts the graph’s ability to highlight the differences in their individual perspective. Yet, their capability to capture interactions at the sentence level enables sentence similarity graphs to learn sentence representations that are contextualized within their surroundings, resulting in a more robust understanding of the interview.

Compared to sentence similarity graphs, KCG representations show greater improvements when combined with multi-view architectures. *KCG-MV* model outperforms *KCG-Baseline* by 11% on macro-F1, 7% on UAR, 12% on Accuracy and 17.6% on Precision (macro). This jump can be attributed to the fact that KCG representations focus on keyword interactions rather than sentence interactions, allowing the model to better integrate view-specific features into the graph structure. Since keyword correlation graphs represent both views as interactions between topical keywords rather than sentences (question-answer interactions in particular), they avoid contextual dependency between the views and learn a more independent representations of the two views. Additionally, the training of dedicated topic models for the two views enhances the independent encoding of view perspectives. This property of KCG is aligned with the multi-view idea resulting in significant performance gain by integrating the two. Although the structural definition of keyword correlation graphs allows them to model corpus-level knowledge into the input graphs that is missing from sentence similarity graphs, they lack in their ability to represent local sentence-level interactions. This lack of local information significantly restricts the learning ability of the model especially for our data where the two views (questions and answers) share a strong contextual bond. This is evident from our results (cf. Table 2) where KCG-based configurations systematically underperform compared to other models, thus showing the strong co-dependence between corresponding questions and answers in defining their meaning. It is important to note that within this paper KCG representations are used only to exemplify different possibilities within graphical input representations and showcase their ability to highlight various aspects of the interview. Results shown for KCG-based inputs use the same configurations as defined by Chiu et al. [18] and have not been tuned to task-specific values. We expect the results to improve with tuned configurations that require detailed research into the various aspects of KCG definition and are left as future work.

Finally, experiments have been carried out with different dense input representations including hierarchical models and BERT-based input embeddings. Among the different combinations and configurations, transformer-based sentence embeddings evidence the best and most stable results. Different implementations for GNNs were also explored including GAT [31] and GIN [21], with all configurations providing similar performance. This behavior is in line with the findings of Dwivedi et al. [32].

## Visualization and insights

Although current deep learning models provide excellent results, explaining their predictions is still a challenging task. Attention scores are widely used as a tool to justify model predictions, however validity of these explanations is debatable [33, 34]. In healthcare applications, despite their high performance, there is a reluctance to adopt black-box neural network models. Instead, medical professionals are more inclined towards models that justify their predictions rather than focusing solely on performance.

Our research not only aims to show the advantages of using graph-based interview representations towards predicting ability of the models, but we also motivate the notion that input representations themselves can be used for insight generation. Our aim is not to provide an explanation of model predictions, but rather use visualizations of the input graphs as a visual summary of the transcripts to be used by medical professionals. These visualizations can highlight information within the transcripts that might be relevant for healthcare professionals, and present it in an easy to comprehend manner. We explore this possibility in the context of sentence similarity and keyword correlation graph structures.

### Sentence similarity and therapist behaviour

**Fig. 4:**
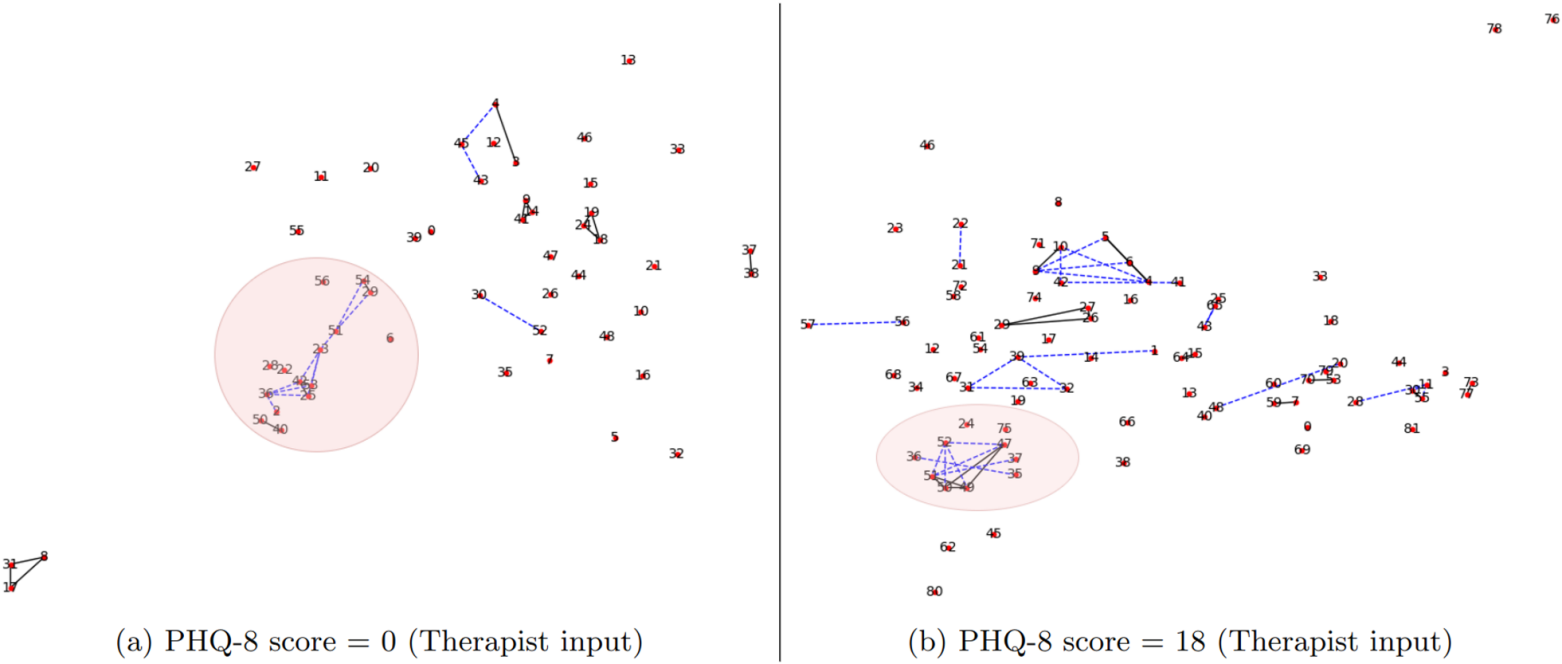
Sentence similarity graphs based on therapist inputs for different PHQ scores. Blue dashed lines represent weak correlations, while black solid lines represent a strong correlation

Figure 4 shows the visualization of sentence similarity graphs based on therapist inputs for patients with different PHQ-8 scores. Each node in the graph represents a question and the numbers are their corresponding position in the input sequence. Clusters highlighted in red comprise of conversation fillers and one-word responses used by the therapist that can be ignored for this analysis. Comparing the remaining clusters, we find more descriptive graphs for people with high depression scores as compared to patients not suffering from depression. People with depression can tend to be more reserved and usually give short and precise answers, forcing the therapist to ask more detailed questions. This is evident from the presence of elaborate clusters within Fig. 4b, where each cluster represents therapist questions regarding relevant aspects of a patient’s life including work, relationships, children, etc. A contrasting view is observed for patients without depression, Fig. 4a, where we see a significant lack of clusters within the graph. This is usually due to the presence of more detailed answers by the patient, allowing the therapist to avoid detailed questions and rely on conversation fillers to sustain the interaction. These visualizations of sentence similarity graphs highlight the subtle differences in therapists’ behavior when interacting with patients having different severity of depression, which in turn can be an indicator of patients’ mental health.

### KCG and global viewpoints

**Fig. 5:**
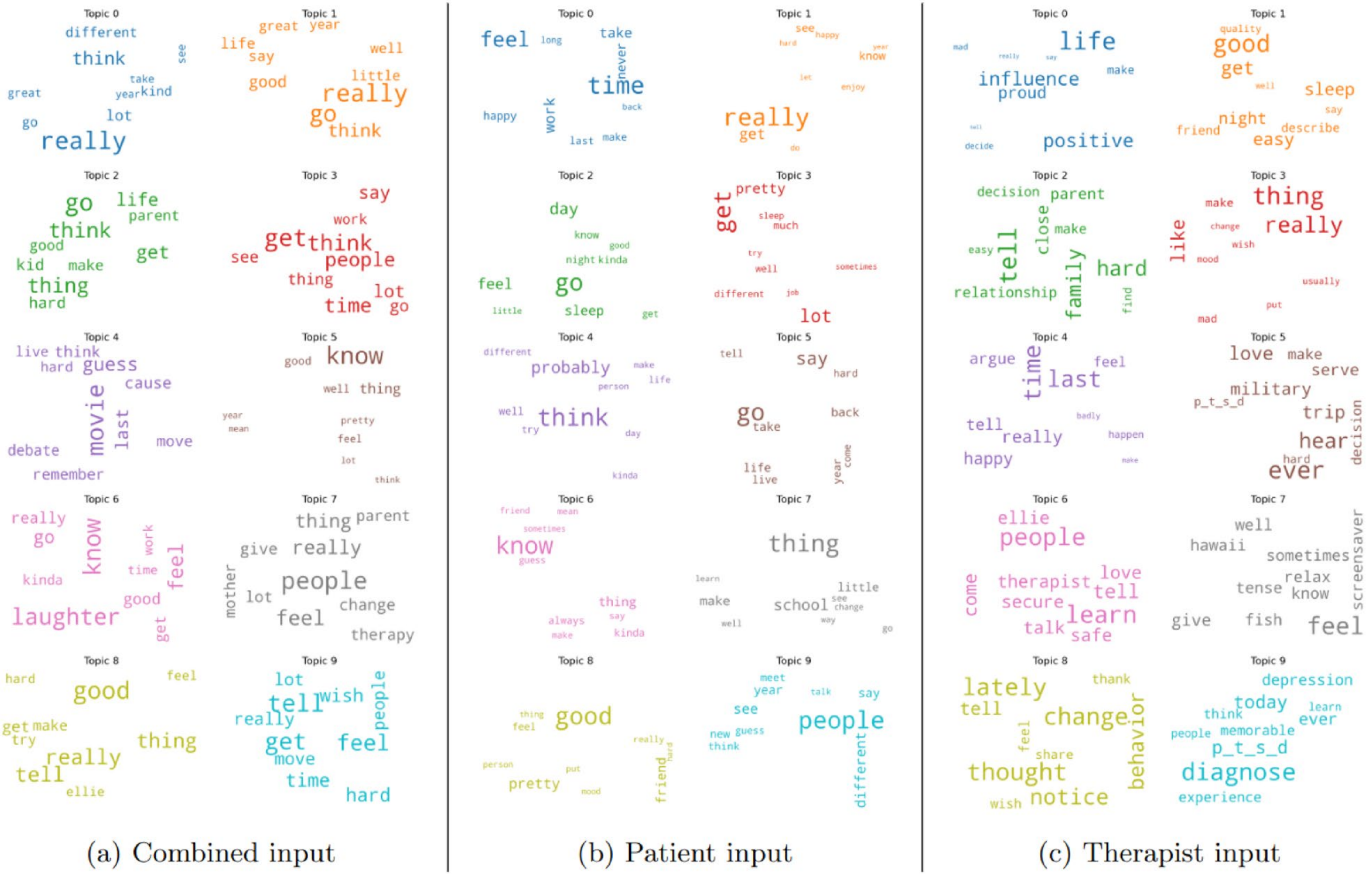
Topics learned with different inputs

Within the context of clinical interviews, patients and therapists have different motives for attending the interview and consequently their respective interventions can provide complementary information about the same discourse. Topic models trained within the KCG definition encapsulate corpus-level knowledge of the input text. Consequently, view-specific topic models more effectively emphasize the differences in perspective between the two agents engaged in an interview compared to a topic model trained on combined input. Figure 5 shows the topics learned within the KCG-Baseline (combined input) and KCG-MV (patient input and therapist input) configurations. In order to understand a patient’s mental health, it is desirable to study the emotions and feelings associated with important aspects of their lives. This involves learning both, the global set of relevant topics (representing important aspects of a person’s life) and patient’s attitude towards them. Comparing the different topic models in Fig. 5, we clearly see that topics based on therapist inputs (Fig. 5c) are better suited for representing the various aspects of a person’s life that have relevance in depression estimation. We see distinct topics representing sleep (topic 1), family (topic 2), positive influence (topic 0), military service (topic 5), change in behavior (topic 8), p_t_s_d and past diagnoses (topic 9), which correlate with the information desired by medical professionals. Within such interviews, therapists usually have a methodical approach toward the interview trajectory, as reflected in the clearly defined topics derived from their inputs. Conversely, patients have a slightly less pronounced role in defining the structure of discourse, primarily responding to topics chosen by the therapist, Fig. 5b. Although topics learned on the combined input, Fig. 5a, contain information on both views, they do not provide a complete knowledge of either and lack specific topics representing important characteristics within each view. These findings highlight the importance of incorporating the discourse structure within the learning process, as proposed by Agarwal et al. [11], rather than treating the input as an unstructured sequence of sentences.

### KCG and transcript level visualization

**Fig. 6:**
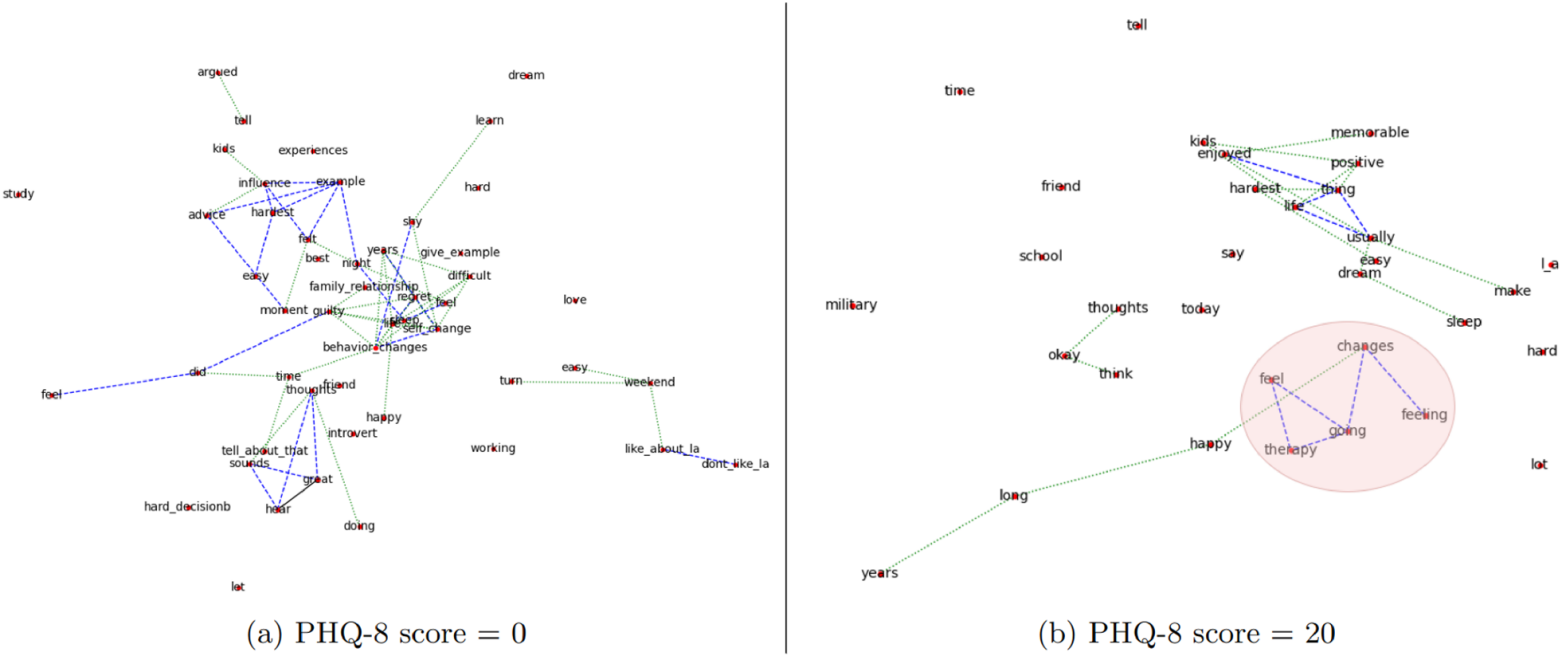
KCG representations of transcripts with different PHQ-8 scores

Another interesting trait of KCG representations can be seen in the visualizations of individual transcript-level graphs. Since these graphs are defined in terms of interactions between the most relevant keywords within individual interviews, their visualization can act as a topical summary of the transcript. Within the context of structured interviews, most global topics are discussed in each transcript albeit with varying importance depending on each patient’s situation. KCG structures utilize this fact by selecting the most important keywords within the transcript to highlight the subtle patterns that can be indicative of patients’ mental health. An example is shown in Fig. 6 that compares graph visualizations for two patients with different depression scores. For patient with high depression score, keywords like therapy, changes and feeling are clustered together (highlighted in red) while being absent from the graph of patient with low depression score. Further analysis of the entire dataset revealed a pattern where keywords like depression, p_t_s_d, and therapy frequently appear as clusters in graphs of patients with high depression scores (score $\ge$ 18) while generally being absent from graphs of non-depressed patients. This highlights the fact that although topics like depression and p_t_s_d are discussed in most interviews, they are more relevant in the context of patients with high depression as compared to those without depression. Consequently, their presence in the graph can be indicative of depressive tendencies and can easily be highlighted within KCG visualizations. These visualizations can illustrate the relative importance of different topics discussed within the interview, which in turn can be an indication of a patient’s mental health.

## Conclusions and future work

In this paper, we explore graph-based representations of patient-therapist interviews as a means to not only exploit the non-linear characteristics of the discourse for improved performance, but also use graph visualizations for generating insights that can prove relevant for medical professionals. Overall results on DAIC-WOZ dataset show advantages of graph-based learning over sequential models in context of binary classification task within automated depression estimation. In particular, graph-based *Similarity-MV* configuration evidences the best performance and provides new state-of-the-art results, thus out-performing comparable sequential configurations [23] and recent works including those relying on external knowledge [9], multiple modalities [10] and multi-target learning [15]. Our experiments on two different graph structures, *sentence similarity graphs* and *keyword correlation graphs*, highlight both, transcript level sentence-sentence interactions and corpus level topical knowledge within the dataset. We further extend the work done by Agarwal et al. [11] by not only incorporating multi-view architectures within graphical models, but also applying the multi-view concept to input representations directly. Figures in Table 2 show systematic improvements with multi-view based configurations across all models considered in our study, thus reinforcing the model-agnostic nature of multi-view concept. We also propose utilization of input representations as a source of insights into the data and use sentence similarity graphs and keyword correlation graphs for our initial experiments in the field. We provide examples in favour of our proposal and show how visualizations of input graph structures can highlight subtle behavioural patterns of patients and therapist that can be indicative of patients mental health.

We plan to continue in this research direction and further explore graphical representation of patient-therapist interviews for generating insights that are relevant for medical professionals working in the field. We intend to delve into a more comprehensive investigation of KCG definitions to enhance the learned topics and fine tune the hyper-parameters for improved predictive performance.

## Data Availability

The DAIC-WOZ data set that supports the findings of this study is available from The University of Southern California Institute for Creative Technologies (https://dcapswoz.ict.usc.edu/) but restrictions apply to the availability of these data, which were used under license for the current study, and so are not publicly available. Data set is, however, available from the University of Southern California Institute for Creative Technologies upon reasonable request [19]. The code of the model will be made available in the author’s repository: https://git.unicaen.fr/navneet.agarwal/graph-based-depression-classification.

## Footnotes

1 https://www.who.int/news-room/fact-sheets/detail/depression.

2 https://www.leprogres.fr/ain-01/2019/03/06/le-departement-en-penurie-de-psychiatres.

## References

[1] Şimşir Z, Koç H, Seki T, Griffiths MD (2022). The relationship between fear of covid-19 and mental health problems: a meta-analysis. Death Stud.

[2] Kroenke K (2012). Enhancing the clinical utility of depression screening. Can Med Assoc J.

[3] Ray A, Kumar S, Reddy R, Mukherjee P, Garg R (2019) Multi-level attention network using text, audio and video for depression prediction. In: 9th international on audio/visual emotion challenge and workshop (AVEC). pp 81–88

[4] Qureshi SA, Saha S, Hasanuzzaman M, Dias G (2019). Multitask representation learning for multimodal estimation of depression level. IEEE Intell Syst.

[5] Qureshi SA, Dias G, Hasanuzzaman M, Saha S (2020). Improving depression level estimation by concurrently learning emotion intensity. IEEE Comput Intell Mag.

[6] Bailey A, Plumbley MD (2021) Gender bias in depression detection using audio features. In: 29th European signal processing conference (EUSIPCO). pp 596–600

[7] Oureshi SA, Dias G, Saha S, Hasanuzzaman M (2021) Gender-aware estimation of depression severity level in a multimodal setting. In: 2021 international joint conference on neural networks (IJCNN), pp 1–8

[8] Mallol-Ragolta A, Zhao Z, Stappen L, Cummins N, Schuller BW (2019) A hierarchical attention network-based approach for depression detection from transcribed clinical interviews. In: Interspeech (INTERSPEECH). pp 221–225

[9] Xezonaki D, Paraskevopoulos G, Potamianos A, Narayanan S (2020) Affective conditioning on hierarchical attention networks applied to depression detection from transcribed clinical interviews. In: Interspeech (INTERSPEECH). pp 4556–4560

[10] Dai Z, Zhou H, Ba Q, Zhou Y, Wang L, Li G (2021). Improving depression prediction using a novel feature selection algorithm coupled with context-aware analysis. J Affect Disord.

[11] Agarwal N, Dias G, Dollfus S Agent-based splitting of patient-therapist interviews for depression estimation. In: Empowering communities: a participatory approach to AI for mental health (PAI4MH) associated to 36th conference on neural information processing systems (NeurIPS)

[12] Niu M, Chen K, Chen Q, Yang L (2021) Hcag: a hierarchical context-aware graph attention model for depression detection. In: IEEE international conference on acoustics, speech and signal processing (ICASSP), pp. 4235–4239

[13] Hong S, Cohn A, Hogg DC (2022) Using graph representation learning with schema encoders to measure the severity of depressive symptoms. In: International conference on learning representations (ICLR)

[14] Burdisso S, Villatoro-Tello E, Madikeri S, Motlicek P (2023) Node-weighted graph convolutional network for depression detection in transcribed clinical interviews. In: Interspeech (INTERSPEECH)

[15] Milintsevich K, Sirts K, Dias G (2023). Towards automatic text-based estimation of depression through symptom prediction. Brain Inform.

[16] Ji S, Zhang T, Ansari L, Fu J, Tiwari P, Cambria E (2022) MentalBERT: Publicly available pretrained language models for mental healthcare. In: 13th language resources and evaluation conference (LREC). pp 7184–7190

[17] Lau C, Zhu X, Chan W-Y (2023). Automatic depression severity assessment with deep learning using parameter-efficient tuning. Front Psychiatry.

[18] Chiu B, Sahu SK, Thomas D, Sengupta N, Mahdy M (2020) Autoencoding keyword correlation graph for document clustering. In: 58th annual meeting of the association for computational linguistics (ACL). pp 3974–3981

[19] Gratch J, Artstein R, Lucas G, Stratou G, Scherer S, Nazarian A, Wood R, Boberg J, DeVault D, Marsella S, et al (2014) The distress analysis interview corpus of human and computer interviews. In: 9th international conference on language resources and evaluation (LREC). pp 3123–3128

[20] Gilmer J, Schoenholz SS, Riley PF, Vinyals O, Dahl GE (2017) Neural message passing for quantum chemistry. In: International conference on machine learning (ICML). pp 1263–1272

[21] Xu K, Hu W, Leskovec J, Jegelka S (2019) How powerful are graph neural networks? In: International conference on learning representations (ICLR)

[22] Wang K, Han SC, Poon J (2022) Induct-gcn: Inductive graph convolutional networks for text classification. In: 26th international conference on pattern recognition (ICPR). pp 1243–1249

[23] Agarwal N, Dias G, Dollfus S (2024) Analysing relevance of discourse structure for improved mental health estimation. In: 9th workshop on computational linguistics and clinical psychology (CLPSYCH) Associated to 18th conference of the European chapter of the association for computational linguistics (EACL)

[24] Févotte C, Idier J (2011). Algorithms for nonnegative matrix factorization with the $$\beta $$-divergence. Neural Comput.

[25] Gratch J, Artstein R, Lucas G, Stratou G, Scherer S, Nazarian A, Wood R, Boberg J, Devault D, Marsella S, Traum D, Rizzo AS, Morency”, L-P (2014) The distress analysis interview corpus of human and computer interviews. In: 9th international conference on language resources and evaluation (LREC) (2014)

[26] DeVault D, Artstein R, Benn G, Dey T, Fast E, Gainer A, Georgila K, Gratch J, Hartholt A, Lhommet M, et al (2014) Simsensei kiosk: A virtual human interviewer for healthcare decision support. In: International conference on autonomous agents and multi-agent systems (AAMAS). pp 1061–1068

[27] Reimers N, Gurevych I (2019) Sentence-BERT: Sentence embeddings using Siamese BERT-networks. In: Conference on empirical methods in natural language processing and the 9th international joint conference on natural language processing (EMNLP-IJCNLP). pp 3982–3992

[28] Kipf TN, Welling M (2017) Semi-supervised classification with graph convolutional networks. In: 5th international conference on learning representations (ICLR)

[29] Vaswani A, Shazeer N, Parmar N, Uszkoreit J, Jones L, Gomez AN, Kaiser Ł, Polosukhin I (2017) Attention is all you need. Advances in neural information processing systems (NeurIPS). 30. https://proceedings.neurips.cc/paper_files/paper/2017/file/3f5ee243547dee91fbd053c1c4a845aa-Paper.pdf

[30] Fey M, Lenssen JE (2019) Fast graph representation learning with PyTorch Geometric. In: ICLR Workshop on representation learning on graphs and manifolds

[31] Velickovic P, Cucurull G, Casanova A, Romero A, Liò P, Bengio Y (2018) Graph attention networks. In: 6th international conference on learning representations (ICLR)

[32] Dwivedi VP, Joshi CK, Luu AT, Laurent T, Bengio Y, Bresson X (2023). Benchmarking graph neural networks. J Mach Learn Res.

[33] Wiegreffe S, Pinter Y (2019) Attention is not not explanation. In: Conference on empirical methods in natural language processing and the 9th international joint conference on natural language processing (EMNLP-IJCNLP). pp 11–20

[34] Jain S, Wallace BC (2019) Attention is not explanation. In: Conference of the North American chapter of the association for computational linguistics: human language technologies (NAACL-HLT)). pp 3543–3556

